# Upregulation of CB_2_ receptors in reactive astrocytes in canine degenerative myelopathy, a disease model of amyotrophic lateral sclerosis

**DOI:** 10.1242/dmm.028373

**Published:** 2017-05-01

**Authors:** María Fernández-Trapero, Francisco Espejo-Porras, Carmen Rodríguez-Cueto, Joan R. Coates, Carmen Pérez-Díaz, Eva de Lago, Javier Fernández-Ruiz

**Affiliations:** 1 Departamento de Bioquímica y Biología Molecular, Instituto Universitario de Investigación en Neuroquímica, Facultad de Medicina, Universidad Complutense, Madrid 28040, Spain; 2 Centro de Investigación Biomédica en Red de Enfermedades Neurodegenerativas (CIBERNED), Madrid 28040, Spain; 3 Instituto Ramón y Cajal de Investigación Sanitaria (IRYCIS), Madrid 28040, Spain; 4 Departamento de Medicina y Cirugía Animal, Facultad de Veterinaria, Universidad Complutense, Madrid 28040, Spain; 5 Department of Veterinary Medicine and Surgery, College of Veterinary Medicine, University of Missouri, Columbia, MO 65211, USA

**Keywords:** Cannabinoids, Endocannabinoid signaling, CB_2_ receptors, Canine degenerative myelopathy, Amyotrophic lateral sclerosis, *SOD1*, Activated astrocytes

## Abstract

Targeting of the CB_2_ receptor results in neuroprotection in the SOD1^G93A^ mutant mouse model of amyotrophic lateral sclerosis (ALS). The neuroprotective effects of CB_2_ receptors are facilitated by their upregulation in the spinal cord of the mutant mice. Here, we investigated whether similar CB_2_ receptor upregulation, as well as parallel changes in other endocannabinoid elements, is evident in the spinal cord of dogs with degenerative myelopathy (DM), caused by mutations in the superoxide dismutase 1 gene (*SOD1*). We used well-characterized post-mortem spinal cords from unaffected and DM-affected dogs. Tissues were used first to confirm the loss of motor neurons using Nissl staining, which was accompanied by glial reactivity (elevated GFAP and Iba-1 immunoreactivity). Next, we investigated possible differences in the expression of endocannabinoid genes measured by qPCR between DM-affected and control dogs. We found no changes in expression of the CB_1_ receptor (confirmed with CB_1_ receptor immunostaining) or NAPE-PLD, DAGL, FAAH and MAGL enzymes. In contrast, CB_2_ receptor levels were significantly elevated in DM-affected dogs determined by qPCR and western blotting, which was confirmed in the grey matter using CB_2_ receptor immunostaining. Using double-labelling immunofluorescence, CB_2_ receptor immunolabelling colocalized with GFAP but not Iba-1, indicating upregulation of CB_2_ receptors on astrocytes in DM-affected dogs. Our results demonstrate a marked upregulation of CB_2_ receptors in the spinal cord in canine DM, which is concentrated in activated astrocytes. Such receptors could be used as a potential target to enhance the neuroprotective effects exerted by these glial cells.

## Abstract (precis)

**Editors' choice:** CB_2_ receptors are upregulated in activated astrocytes recruited at the damaged spinal cord in dogs with degenerative myelopathy, a canine model of amyotrophic lateral sclerosis.

## INTRODUCTION

Amyotrophic lateral sclerosis (ALS) is a progressive degeneration and loss of upper and lower motor neurons in the brain and spinal cord, causing muscle weakness and paralysis (Hardiman et al., 2011). In 1993, genetic studies identified the first mutations in the copper-zinc superoxide dismutase gene (*SOD1*), which encodes a key antioxidant enzyme, SOD1 (Rosen et al., 1993). Mutations in *SOD1* account for 20% of genetic ALS and 2% of all ALS. More recently, similar studies have identified mutations in other genes, such as *TARDBP* (TAR-DNA binding protein) and *FUS* (fused in sarcoma), which encode proteins involved in pre-mRNA splicing, transport and/or stability (Buratti and Baralle, 2010; Lagier-Tourenne et al., 2010) and, in particular, the CCGGGG hexanucleotide expansion in the *C9orf72* gene, which appears to account for up to 40% of genetic cases (Cruts et al., 2013). Their pathogenic mechanisms, which differ, in part, from the toxicity associated with mutations in SOD1, led to a novel molecular classification of ALS subtypes (Al-Chalabi and Hardiman, 2013; Renton et al., 2014).

The ultimate goal in ALS is to develop novel therapeutics that will slow disease progression. Rilutek has been the only drug approved by the US Food and Drug Administration (FDA), but it is limited in efficacy (Habib and Mitsumoto, 2011). Recently, cannabinoids have been shown to have neuroprotective effects in transgenic rodent ALS models (Bilsland and Greensmith, 2008; de Lago et al., 2015, for review). Chronic treatment with the phytocannabinoid Δ^9^-tetrahydrocannabinol (Δ^9^-THC) delayed motor impairment and improved survival in the SOD-1^G93A^ transgenic mouse (Raman et al., 2004). Other cannabinoid compounds, including the less psychotropic plant-derived cannabinoid cannabinol (Weydt et al., 2005), the non-selective synthetic agonist WIN55,212-2 (Bilsland et al., 2006), and the selective cannabinoid receptor type-2 (CB_2_) agonist AM1241 (Kim et al., 2006; Shoemaker et al., 2007), produced similar effects. Genetic or pharmacological inhibition of fatty acid amide hydrolase (FAAH), one of the key enzymes in endocannabinoid degradation, was also beneficial in SOD1^G93A^ transgenic mice (Bilsland et al., 2006). The efficacy shown by compounds that target the CB_2_ receptor (Kim et al., 2006; Shoemaker et al., 2007) appears to be facilitated by the fact that this receptor is upregulated in reactive glia in post-mortem spinal cord tissue from ALS patients (Yiangou et al., 2006). Such elevation of CB_2_ receptors has been also described in SOD1^G93A^ transgenic mice (Shoemaker et al., 2007; Moreno-Martet et al., 2014), and we recently found that the response occurred predominantly in activated astrocytes recruited at lesion sites in the spinal cord (F.E.P., unpublished results). We have also described a similar increase in CB_2_ receptors on reactive microglia in TDP-43 transgenic mice (Espejo-Porras et al., 2015). Based on these studies, the CB_2_ receptor may be a novel target in altering disease progression in ALS, given its effective control of glial influences exerted on neurons, as investigated in other disorders (Fernández-Ruiz et al., 2007, 2015; Iannotti et al., 2016 for review).

A challenge of preclinical studies of novel neuroprotective agents in ALS is poor translation of therapeutic success in small animal (e.g. rodents, zebrafish, flies, nematodes) to human ALS patients. Most studies have been based on overexpression of specific human gene mutations. In this context, we have recently turned to canine degenerative myelopathy (DM), a multisystem central and peripheral axonopathy described in dogs in 1973 (Averill, 1973), with an overall prevalence of 0.19% (Coates and Wininger, 2010 for review), which shares pathogenic mechanisms with some forms of human ALS, including mutations in SOD1 as one of the major causes of the disease (Awano et al., 2009). With some differences depending on the type of breed, DM is characterized by degeneration in the white matter of the spinal cord and the peripheral nerves, which progresses to affect both upper and lower motor neurons (Coates and Wininger, 2010 for review). The disease appears at 8-14 years of age with an equivalent effect in both sexes that necessitates euthanasia (Coates and Wininger, 2010 for review). This canine pathology represents a unique opportunity to investigate ALS in a context much closer to the human pathology, using an animal species that are phylogenetically closest to humans, and in which the disease occurs spontaneously. Our objective in the present study has been to investigate the changes that the development of DM produces in endocannabinoid elements in those CNS sites (spinal cord) most affected in this disease. It is important to note that such elements may result in potential targets for a pharmacological therapy with cannabinoid-based therapies (e.g. Sativex) aimed at delaying/arresting the progression of the disease in these dogs, and ultimately in humans. The study was carried out with post-mortem tissues (spinal cords) from dogs affected by DM kindly provided by Dr Joan R. Coates (University of Missouri, Columbia, MO, USA) and classified in different disease stages (Coates and Wininger, 2010). All DM tissues included the necessary clinical, genetic and neuropathological information, and they were accompanied by adequate matched control tissues. Both DM-affected and control tissues were used for analysis of endocannabinoid receptors and enzymes using biochemical (qPCR, western blot) and, in some cases, histological (immunohistochemistry) procedures, including the use of double immunofluorescence staining to identify the cellular substrates in which the changes in endocannabinoid elements (CB_2_ receptors) take place.

## RESULTS

### Validation of the expected histopathological deterioration in DM-affected dogs

The data provided by the biobank confirmed that all tissues obtained from DM-affected dogs had a clinical diagnosis of DM in all cases supported by the genetic analysis which confirmed the presence of the SOD1 mutation. They corresponded to two different breeds, which are from species most affected by this disease (Coates and Wininger, 2010), and animals were all euthanized in an age interval of 9-13.6 years (11.8±0.6; mean±s.d.), with a grade of the disease of 1-3 (2.2±0.3). DM-affected dogs included 6 spayed females and 2 castrated males (see details in Table 1). The control tissues were selected from dogs with no clinical diagnosis of DM. All control dogs were homozygous wild-type and age-matched (8-13.6 years; 10.0±0.8). They included 6 females, 1 of them spayed, and 1 castrated male (see details in Table 1).

**Table 1.**
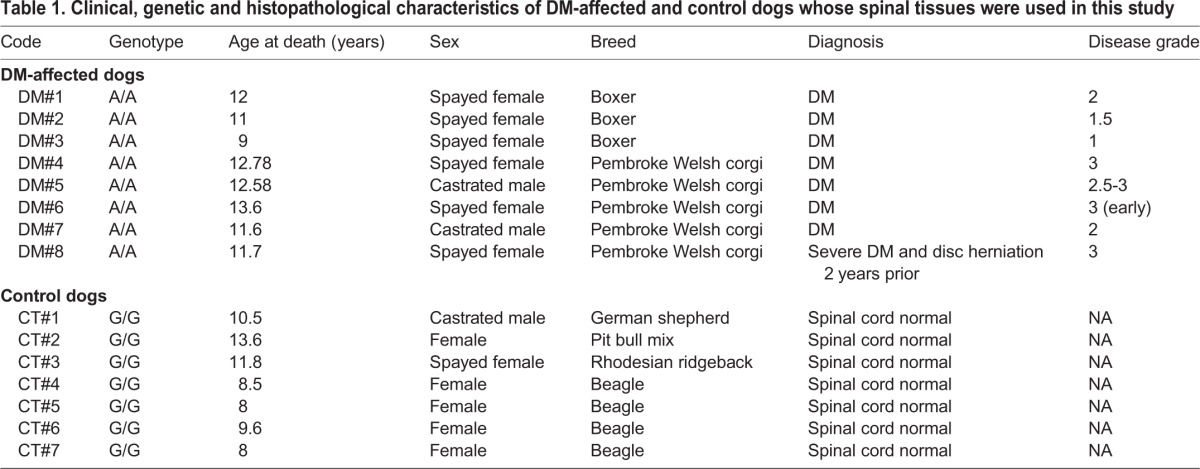
**Clinical, genetic and histopathological characteristics of DM-affected and control dogs whose spinal tissues were used in this study**

**Fig. 1.**
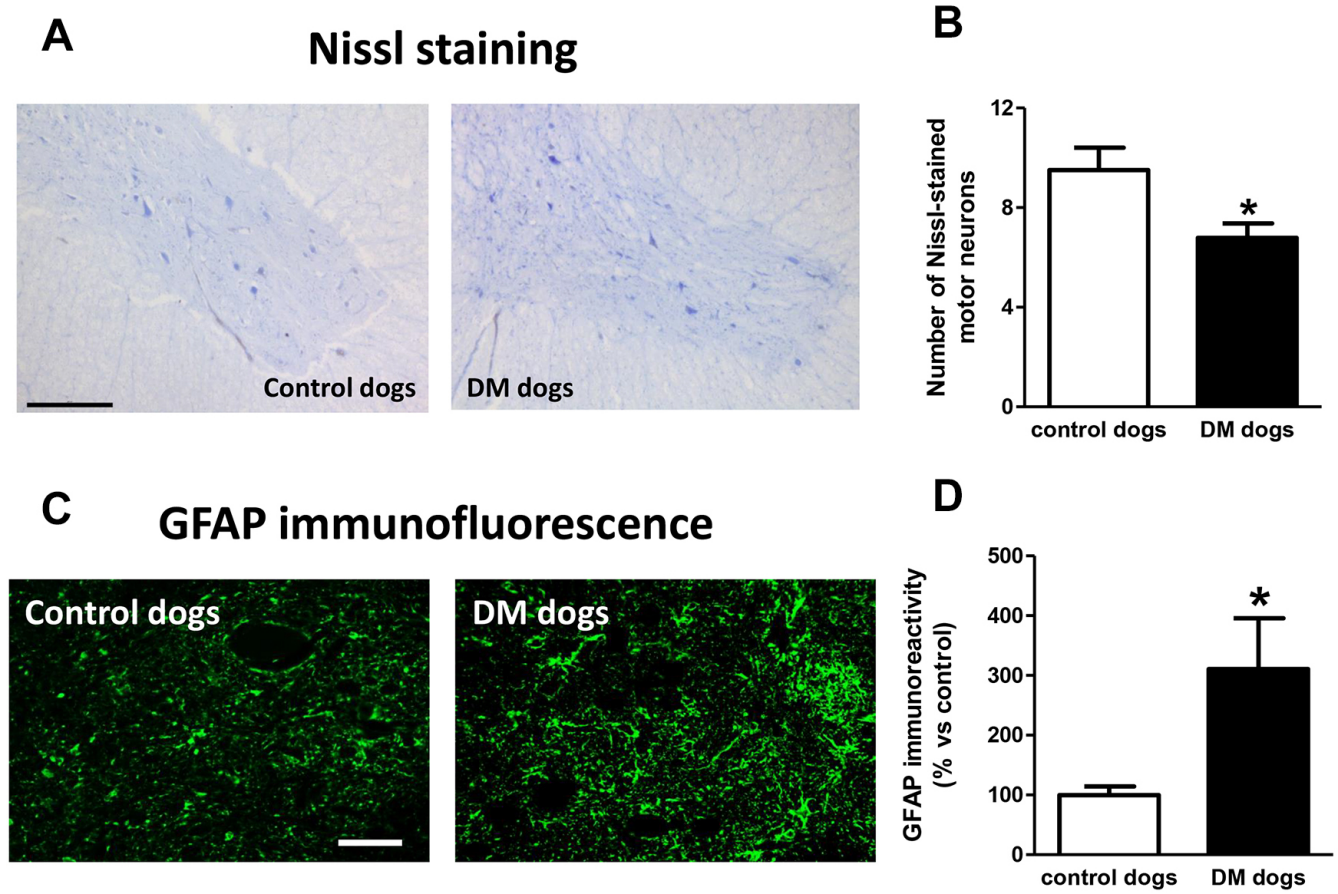
**Nissl staining and glial activity in spinal cord sections of dogs with degenerative myelopathy (DM).** Representative photomicrographs and quantification of Nissl staining (A,B) and GFAP immunofluorescence (C,D) in spinal cord sections (grey matter in the ventral horn at T7-T10) of DM-affected and age-matched control dogs. Values are expressed as means±s.e.m. for 6-7 animals per group. Data were analysed using the unpaired Student's *t*-test (*P<0.05 compared with control animals). Scale bars: 300 µm (A) and 200 µm (C).

**Fig. 2.**
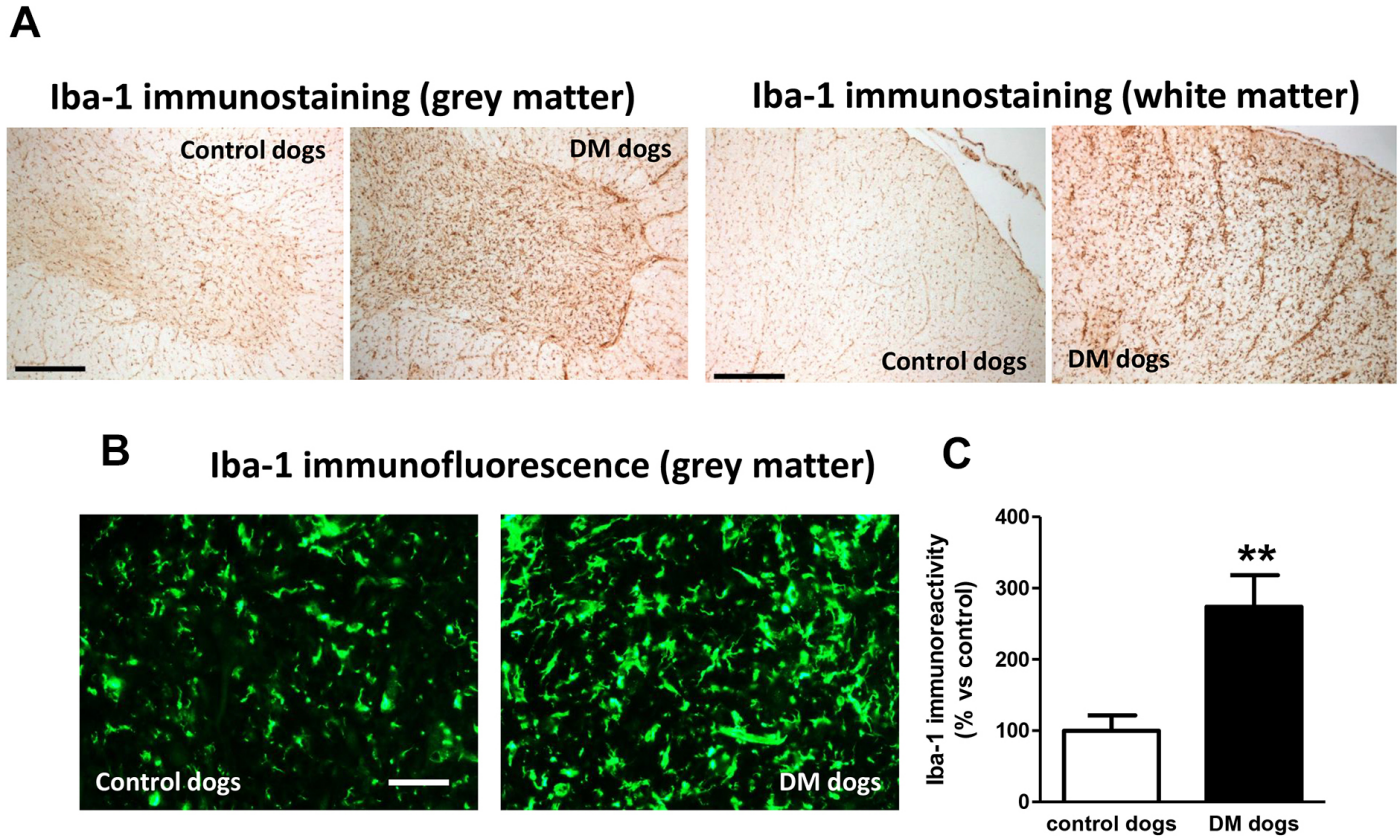
**Iba-1 distribution in spinal cord sections of DM-affected dogs.** Representative photomicrographs of Iba-1 immunostaining using DAB (A) and Iba-1 immunofluorescence (B) and its quantification (C) in spinal cord sections (grey matter in the ventral horn and white matter in the dorsal area, both at T7-T10) of DM-affected and age-matched control dogs. Values are expressed as means±s.e.m. for 5-7 animals per group. Data were analysed using the unpaired Student's *t*-test (**P<0.01 compared with control animals). Scale bar: 300 µm (A) and 200 µm (B).

We found a significant reduction in the number of Nissl-stained cell bodies corresponding to lower motor neurons located in the ventral horn of DM-affected spinal cords (Fig. 1A,B). The neuronal loss was accompanied by an intense glial reactivity in the affected areas, in particular, we detected a 3-fold increase in GFAP immunolabelling in the spinal grey matter (Fig. 1C,D). We also found microgliosis in both white and grey matter of the spinal cord, detected with DAB immunostaining for the microglial marker Iba-1 (Fig. 2A); Iba-1 levels were increased 2.5-fold in the grey matter of DM-affected dogs compared with levels in control dogs (Fig. 2B,C).

### Changes in the endocannabinoid receptors and enzymes in DM-affected dogs

**Fig. 3.**
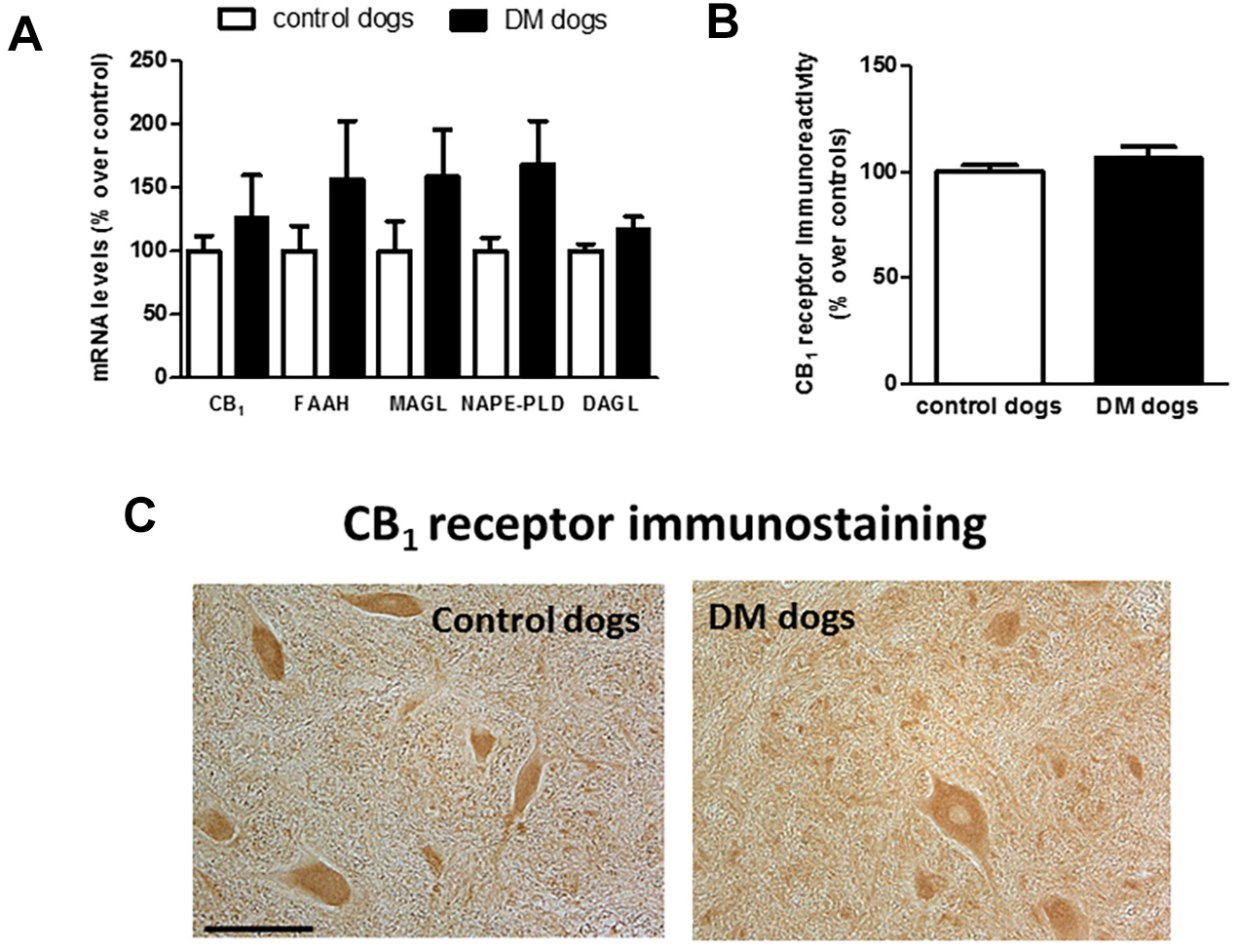
**Status of CB_1_ receptors in spinal samples of DM-affected dogs.** Gene expression for the CB_1_ receptor (*CNR1*) and *NAPE-PLD*, *DAGL*, *FAAH* and *MAGL* measured by qPCR (A), and representative microphotographs for CB_1_ receptor immunostaining using DAB (C) and its quantification in the grey matter in the ventral horn (B), in the spinal cord samples (for qPCR) or T7-T10 sections (for immunostaining) of DM-affected and age-matched control dogs. Values are expressed as means±s.e.m. for 7-8 animals per group. Data were analysed using the unpaired Student's *t*-test. Scale bar: 150 µm.

Next, we investigated possible differences between DM-affected dogs and control animals in the expression of endocannabinoid genes measured by qPCR. Although there was a trend towards an elevation, there were no significant changes in expression of the CB_1_ receptor (*CNR1*), or *FAAH*, monoacylglcerol lipase (*MAGL*), *N*-arachidonoyl-phosphatidylethanolamine phospholipase D (*NAPE-PLD*) and diacylglycerol lipase (*DAGL*) enzymes between the two groups (Fig. 3A). We attempted to determine whether the trends detected for these five parameters may correspond to a greater effect in DM dogs with advanced disease, but we did not find any statistically significant correlation (data not shown). They were not related to gender-dependent differences (data not shown). The absence of changes in CB_1_ receptor gene expression was observed at the protein level using DAB immunostaining in the grey matter (Fig. 3B,C). This occurred despite the reduction in the number of motor neurons detected with Nissl staining.

**Fig. 4.**
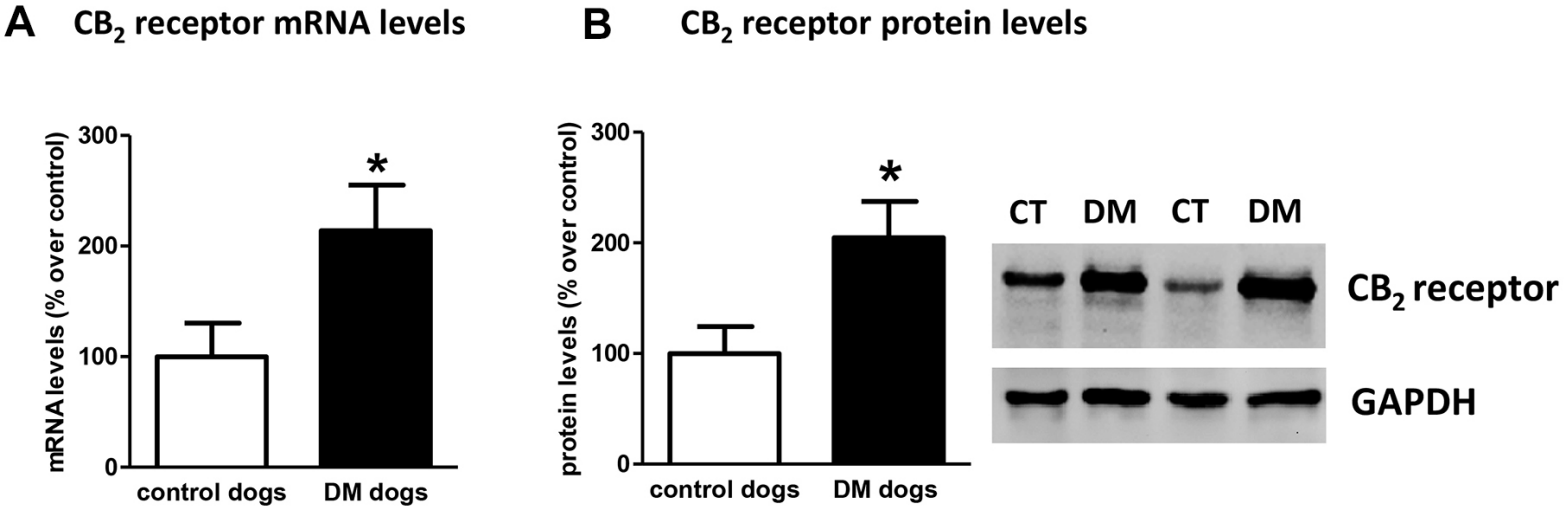
**Gene expression and protein levels of CB_2_ receptor in DM-affected dogs.** Gene expression of the CB_2_ receptor (*CNR2*) measured by qPCR (A), as well as western blot analysis for this receptor (B) in spinal cord samples of DM-affected and age-matched control dogs. Values correspond to % over control animals and are expressed as means±s.e.m. for 7 animals per group. Data were analysed using the unpaired Student's *t*-test (*P<0.05 compared with control animals).

**Fig. 5.**
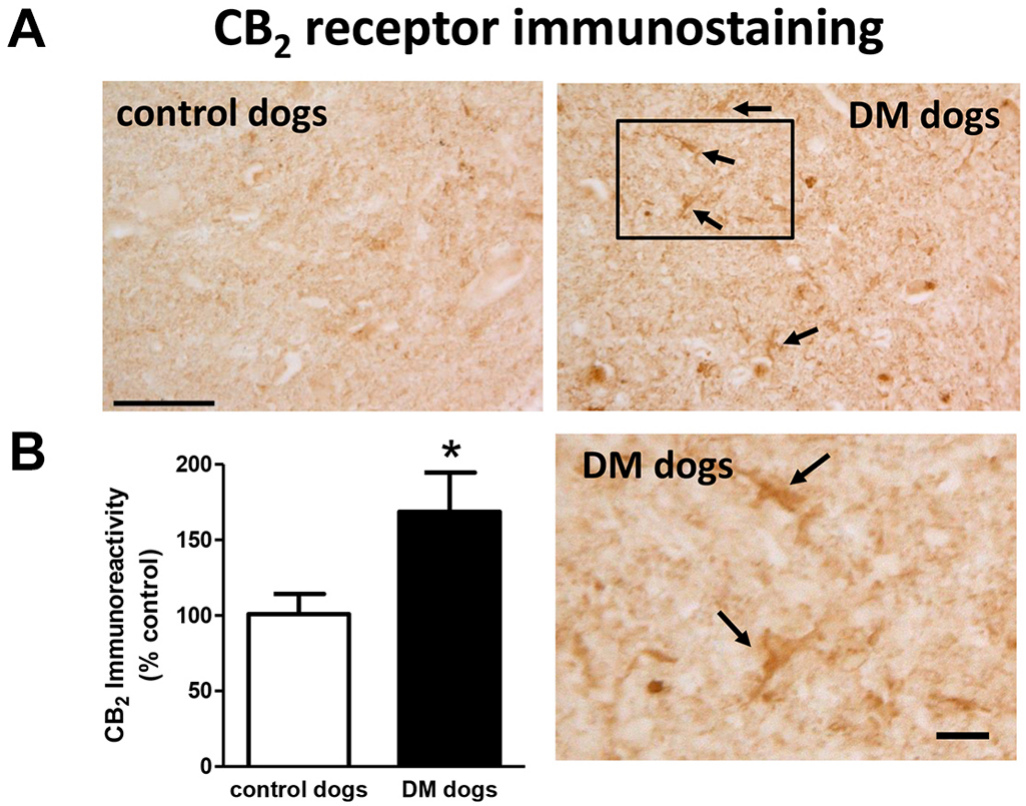
**CB_2_ receptor immunostaining and quantification in DM-affected dogs.** Representative photomicrographs for CB_2_ receptor immunostaining using DAB (A) and its quantification (B) in the grey matter of the ventral horn in T7-T10 spinal cord sections of DM-affected and age-matched control dogs. Values are expressed as means±s.e.m. for 5-6 animals per group. Boxed region in DM dog image is shown enlarged in panel below. Data were analysed using the unpaired Student's *t*-test (*P<0.05 compared with control animals). Scale bars: 150 µm and 50 µm (enlargement). Arrows indicate CB_2_ receptor-positive cells.

Next, we investigated the CB_2_ receptor, an endocannabinoid element that is frequently altered in conditions of neurodegeneration (Fernández-Ruiz et al., 2007, 2015; Iannotti et al., 2016), including ALS (Yiangou et al., 2006; Shoemaker et al., 2007; Moreno-Martet et al., 2014; Espejo-Porras et al., 2015). First, we detected an increase of more than 2-fold in CB_2_ receptor (*CNR2*) expression, measured by qPCR, in DM-affected dogs (Fig. 4A). We also investigated whether this increase occurred predominantly in the tissues obtained from DM-affected dogs at the intermediate and advanced stages, but we did not find any significant correlation between both variables (data not shown). This increase in gene expression was confirmed at the protein level using western blotting (2-fold increase; Fig. 4B), as well as using DAB immunostaining, which showed that the number of CB_2_ receptors increased predominantly in the grey matter (Fig. 5A,B).

### Double-labelling analyses to identify the CB_2_ receptor-positive cellular substrates

**Fig. 6.**
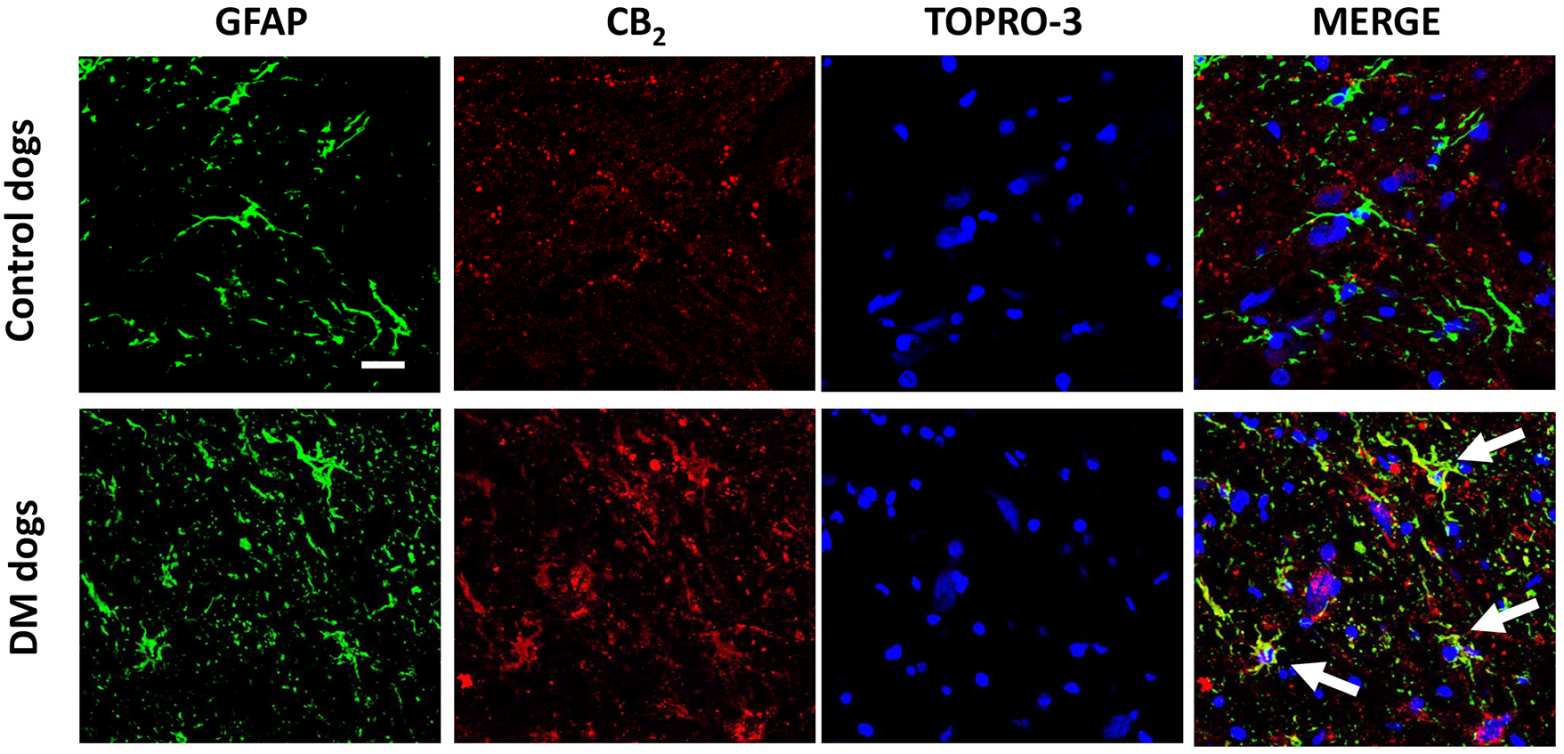
**Double immunofluorescence analysis for CB_2_ receptor and GFAP in spinal cord sections of DM-affected dogs.** Representative photomicrographs showing double immunofluorescence analysis for the CB_2_ receptor and GFAP, using TOPRO-3 for labelling cell nuclei, in the grey matter of the ventral horn in T7-T10 spinal cord sections of DM-affected and age-matched control dogs (*n*=3/group). Scale bar: 50 µm. Arrows indicate cells labelled with the antibodies for the two markers.

**Fig. 7.**
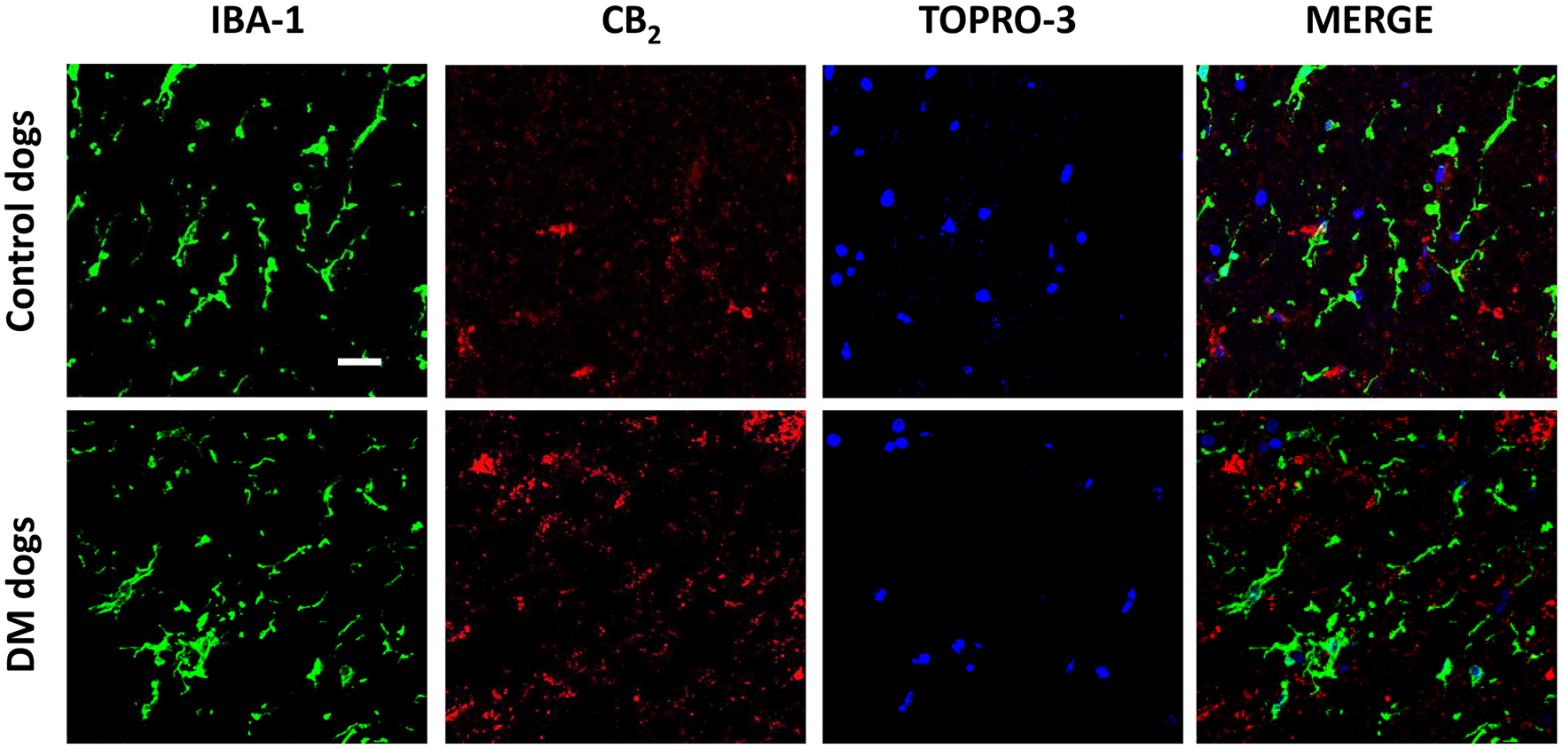
**Double immunofluorescence analysis for CB_2_ receptor and Iba-1 in DM-affected dogs.** Representative photomicrographs showing double immunofluorescence analysis for the CB_2_ receptor and Iba-1, using TOPRO-3 for labelling cell nuclei, in the grey matter of the ventral horn in T7-T10 spinal cord sections of DM-affected and age-matched control dogs (*n*=3/group). Scale bar: 50 µm.

Examination of the morphology of those cells positive for the CB_2_ receptor in DAB immunostaining (Fig. 5A) suggested they were glial cells. We wanted to confirm this by using double-labelling immunofluorescence analysis. We found that CB_2_ receptor immunolabelling colocalized with GFAP immunofluorescence (Fig. 6), thus indicating that the upregulation of CB_2_ receptors in the spinal cord of DM-affected dogs occurred in reactive astrocytes. Similar double-labelling immunofluorescence with Iba-1 did not detect any colocalization with the CB_2_ receptor immunostaining, indicating that the receptor is not located in microglial cells in the spinal cord of DM-affected dogs (Fig. 7).

## DISCUSSION

Our study addressed changes in specific endocannabinoid elements in canine DM, a disease of older dogs with similarities to ALS (Coates and Wininger, 2010 for review). The endocannabinoid system has been previously investigated in different regions of the canine brain (Pirone et al., 2016), but this is the first time that these elements have been investigated in the context of an important neurodegenerative disorder occurring in dogs. The benefits of such an investigation could result in the development of cannabinoid-based therapies for human ALS, but these studies may also serve as a first step in a cannabinoid-based pharmacotherapy useful for veterinary medicine. Our study has investigated the six endocannabinoid elements commonly recognized to develop pharmacological therapies, and has identified the CB_2_ receptor as promising potential target. It is important to mention that our study demonstrates no loss of CB_1_ receptors, which are typically located in neurons, despite the loss of motor neurons occurring in canine DM. This suggests that, contrary to other neurodegenerative conditions in humans, which suffer a profound loss of neuronal CB_1_ receptors, e.g. Huntington's disease (Fernández-Ruiz et al., 2015), the CB_1_ receptor may serve as a potential target in canine DM (shown here) and also in human ALS (de Lago et al., 2015).

As we report for canine DM here, the CB_2_ receptor also becomes strongly upregulated in activated glia in response to neuronal damage in transgenic ALS rodent models (Shoemaker et al., 2007; Moreno-Martet et al., 2014; Espejo-Porras et al., 2015) and ALS patients (Yiangou et al., 2006). The response is not exclusive to ALS but is also observed in other acute or chronic neurodegenerative disorders (e.g. ischemia, Alzheimer's disease, Parkinson's disease, Huntington's disease; reviewed in Fernández-Ruiz et al., 2007, 2015; Iannotti et al., 2016). These findings support the idea that the rise in CB_2_ receptors in activated glial elements is an endogenous response of endocannabinoid signalling aimed at protecting neurons against cytotoxic insults, as well as restoring neuronal homeostasis and integrity (Pacher and Mechoulam, 2011; de Lago et al., 2015; Fernández-Ruiz et al., 2015).

Results of our study further demonstrated that the elevation of CB_2_ receptors occurs in activated astrocytes rather than in microglial cells. This finding has been previously described in the spinal cords of transgenic SOD1 mice (F.E.P., unpublished results). In other transgenic models of ALS, e.g. TDP-43 transgenic mice, the upregulatory response of these receptors occurs predominantly in reactive microglial cells (Espejo-Porras et al., 2015) and in tissues of human ALS patients (Yiangou et al., 2006). In multiple sclerosis and Huntington's disease, the overexpression of CB_2_ receptors occurs in both activated astrocytes and reactive microglia (Benito et al., 2007; Sagredo et al., 2009). The increase in CB_2_ receptors in activated glial elements may be related to the influence of these cells on neuronal homeostasis, for example, by enhancing metabolic support and glutamate reuptake activity exerted by astrocytes (Fernández-Ruiz et al., 2015 for review), by facilitating the transfer of microglial cells from M1 to M2 phenotypes (Mecha et al., 2016 for review) or by attenuating the generation of proinflammatory cytokines, chemokines, nitric oxide and reactive oxygen species by either astrocytes or microglial cells when they become activated (Fernández-Ruiz et al., 2015 for review). These possibilities place the receptor in a promising position for the development of novel therapies. In light of our present study, and given their preferential location in activated astrocytes, we will need to conduct additional research aimed at investigating the consequences of selective CB_2_ receptor activation in these glial cells during the progression of this canine disease.

In conclusion, our results demonstrated a marked upregulation of CB_2_ receptors occurring in the spinal cord of dogs affected by DM. Such upregulation occurred in the absence of changes in other endocannabinoid elements and was concentrated in activated astrocytes, then becoming a potential target to enhance the protective effects exerted by these glial cells to improve neuronal homeostasis and integrity.

## MATERIALS AND METHODS

### Management of the post-mortem tissues

All experiments were conducted on post-mortem spinal cord tissues collected from DM-affected and unaffected dogs. All tissues (formalin-fixed tissues for routine histopathology and frozen tissues for qPCR and western blotting) were provided by Dr Joan R. Coates (Department of Veterinary Medicine and Surgery, College of Veterinary Medicine, University of Missouri, Columbia, MO, USA). Protocols for tissue collection were approved by the University of Missouri Animal Care and Use Committee.

Tissues provided included those of DM-affected dogs and age-matched controls and accompanied by adequate clinical and genetic testing information (see details in Table 1). DM diagnoses were confirmed histopathologically by assessing the mid to lower thoracic spinal cord segment for evidence of myelinated axon loss and pronounced astrogliosis in the dorsal portion of the lateral funiculus (Averill, 1973; March et al., 2009). Dogs that had exhibited clinical signs of DM but did not show the typical histopathology were presumed to have another cause for the myelopathy and were excluded from the study. The spinal cord segments were examined for the presence of SOD1-immunoreactive aggregates within ventral horn motor neurons (Avano et al., 2009). Dogs that had not exhibited any clinical signs of DM prior to euthanasia and whose thoracic spinal cords were histologically normal were used as controls.

Tissues from DM-affected dogs were sorted by different stages of disease progression characterized at origin according to the following clinical and histopathological characteristics: (i) Stage 1 (upper motor neuron paraparesis): progressive general propioceptive ataxia and asymmetric spastic paraparesis, but intact spinal reflexes; (ii) Stage 2 (non-ambulatory paraparesis to paraplegia): mild to moderate loss of muscle mass, reduced to absent spinal reflexes in pelvic limbs, and possible urinary and faecal incontinence; (iii) Stage 3 (lower motor neuron paraplegia to thoracic limb paresis): signs of thoracic limb paresis, flaccid paraplegia, severe loss of muscle mass in pelvic limbs, and urinary and faecal incontinence; and (iv) Stage 4 (lower motor neuron tetraplegia and brainstem signs): flaccid tetraplegia, difficulty with swallowing and tongue movements, reduced to absent cutaneous trunci reflex, generalized and severe loss of muscle mass, and urinary and faecal incontinence (see details in Coates and Wininger, 2010). All DM tissues were accompanied by details of symptoms, genotype and clinical diagnosis (see details in Table 1). Tissue studies confirm the loss of motor neurons using Nissl staining and accompanied by analysis of glial reactivity using GFAP and Iba-1 immunostaining. Next, we investigated the status of endocannabinoid receptors and enzymes using biochemical (qPCR, western blot) and, in some cases, immunostaining procedures, including double immunofluorescence staining to identify the cellular substrates in endocannabinoid elements (CB_2_ receptors). For all measures, tissues used corresponded to 7-8 different animals per experimental group.

### Real-time qRT-PCR analysis

Total RNA was extracted from spinal cord samples (from T7 to T10) using TRI Reagent (Sigma). The total amount of RNA extracted was quantified by spectrometry at 260 nm and its purity was evaluated by the ratio between the absorbance values at 260 and 280 nm, whereas its integrity was confirmed in agarose gels. To prevent genomic DNA contamination, DNA was removed and single-stranded complementary DNA was synthesized from 0.6 μg total RNA using a commercial kit (RNeasy Mini Quantitect Reverse Transcription, Qiagen). The reaction mixture was kept frozen at −80°C until enzymatic amplification. Quantitative real-time PCR assays were performed using TaqMan Gene Expression Assays (Applied Biosystems) to quantify mRNA levels for CB_1_ receptor (Cf02716352_u1), CB_2_ receptor (Cf02696139_s1), DAGL (Cf02705627_m1), FAAH (Cf02648944_m1) and MAGL (Cf02662432_m1). For NAPE-PLD, we used a custom-designed assay (Custom Plus TaqMan RNA Assay Design, Applied Biosystems). In all cases, we used GAPDH expression (Cf04419463_gH) as an endogenous control gene for normalization. The PCR assay was performed using the 7300 Fast Real-Time PCR System (Applied Biosystems) and the threshold cycle (Ct) was calculated by the instrument's software (7300 Fast System, Applied Biosystems). Values were normalized as percentages over the control group.

### Western blot analysis

Purified protein fractions were isolated using ice-cold RIPA buffer. Then, 20 μg protein was boiled for 5 min in Laemmli SDS loading buffer (10% glycerol, 5% SDS, 5% β-mercaptoethanol, 0.01% Bromophenol Blue and 125 mM Tris-HCl, pH 6.8) and loaded onto a 12% acrylamide gel (Bio-Rad), and then transferred to a PVDF membrane (Immobilon-P, Millipore) using mini Trans-Blot Electrophoretic transfer cell (Bio-Rad). Membranes were blocked with 5% non-fat milk and incubated overnight at 4°C with a mouse anti-CB_2_ receptor antibody (1:200; Santa Cruz Biotechnology, SC-293188), followed by a second incubation during 2 h at room temperature with an ECL horseradish peroxidase-linked whole secondary antibody (GE Healthcare) at a 1:5000 dilution. Reactive bands were detected by chemiluminescence with the Amersham ECL Prime Western Blotting Detection Reagent (GE Healthcare). Images were analysed on a ChemiDoc station with Quantity One software (Bio-Rad). Data were calculated as the ratio between the optical densities of the specific protein band and the housekeeping protein GAPDH, and they were normalized as percentages over the control group.

### Histological procedures

#### Tissue slicing

Fixed spinal cords were sliced with a cryostat at the thoracic level, always between T7-T10, which correspond to the spinal level in which the axonal degeneration was most severe (Coates and Wininger, 2010). Coronal sections (20 μm thick) were collected on gelatin-coated slides. Sections were used for procedures of Nissl-staining, immunohistochemistry and immunofluorescence.

#### Nissl staining

Slices were used for Nissl staining using Cresyl Violet, as previously described (Alvarez et al., 2008). A Leica DMRB microscope (Leica, Wetzlar, Germany) and a DFC300FX camera (Leica) were used for the observation and photography of the slides, respectively. For counting the number of Nissl-stained large motor neurons in the anterior horn, high-resolution photomicrographs were taken with the 20× objective under the same conditions of light, brightness and contrast. Four images coming from at least three sections per animal were analysed. The final value is the mean for all animals included in each experimental group.

#### Immunohistochemistry

Slices were pre-incubated for 20 min in 0.1 M PBS with 0.1% Triton X-100, pH 7.4, and subjected to endogenous peroxidase blockade by incubation for 1 h at room temperature in peroxidase blocking solution (Dako). Then, they were incubated in 0.1 M PBS with 0.01% Triton X-100, pH 7.4, with one of the following primary antibodies: (i) polyclonal anti-rabbit Iba-1 antibody (1:500; Wako Chemicals, #019-19741); (ii) polyclonal anti-rabbit CB_1_ receptor (1:400; Thermo Fisher Scientific, PA1-743) and (iii) polyclonal anti-goat CB_2_ receptor antibody (1:100; Santa Cruz Biotechnology, SC10076). Samples were incubated overnight at 4°C, then sections were washed in 0.1 M PBS and incubated for 2 h at room temperature with the appropriate biotin-conjugated anti-goat or anti-rabbit (1:200; Vector Laboratories) secondary antibodies. The Vectastain Elite ABC kit (Vector Laboratories) and a DAB substrate-chromogen system (Dako) were used to obtain a visible reaction product. Negative control sections were obtained using the same protocol with omission of the primary antibody. All sections for each immunohistochemical procedure were processed at the same time and under the same conditions. A Leica DMRB microscope (Leica, Wetzlar, Germany) and a DFC300FX camera (Leica) were used for slide observation and photography.

#### Immunofluorescence

Quantification of GFAP and Iba-1 immunoreactivity was also carried out using immunofluorescence, and this procedure was also used for double-labelling studies. Slices were preincubated for 1 h with Tris-buffered saline with 1% Triton X-100 (pH 7.5). Then, sections were sequentially incubated overnight at 4°C with a polyclonal anti-Iba-1 (1:500; Wako Chemicals, #019-19741) or polyclonal anti-GFAP (1:200; Dako, Z0334), followed by washing in Tris-buffered saline and a further incubation (at 37°C for 2 h) with an Alexa Fluor 488 anti-rabbit antibody conjugate made in donkey (1:200; Invitrogen), rendering green fluorescence for anti-Iba-1 or anti-GFAP. Immunofluorescence was quantified using a SP5 Leica confocal microscope and ImageJ software (US National Institutes of Health, Bethesda, Maryland, USA). For double-labelling studies, sections were then washed again and incubated overnight at 4°C with a polyclonal anti-CB_2_ receptor (1:100; Santa Cruz Biotechnology, SC-10076). This was followed by washing in Tris-buffered saline and a further incubation (at room temperature for 2 h) with a biotin-conjugated anti-goat (1:200; Vector Laboratories) secondary antibody, followed by a new washing and an incubation (at 37°C for 2 h) with red streptavidin (Vector Laboratories, Burlingame, CA, USA) rendering red fluorescence for anti-CB_2_ receptor. Sections were counter-stained with nuclear stain TOPRO-3-iodide (Molecular Probes) to visualize cell nuclei. To quench endogenous autofluorescence, tissue sections were also treated with 0.5% Sudan Black (Merck, Darmstadt, Germany) in 70% ethanol for 1 min and differentiated with 70% ethanol (Schnell et al., 1999). A Leica TCS SP5 microscope was used for slide observation and photography. Differential visualization of the fluorophores was accomplished through the use of specific filter combinations. Samples were scanned sequentially to avoid any potential for bleed-through of fluorophores.

### Statistics

Data were assessed by unpaired Student's *t*-test or one-way ANOVA followed by the Student-Newman-Keuls test, as required.
